# Interleukin-6 Modulation of Intestinal Epithelial Tight Junction Permeability Is Mediated by JNK Pathway Activation of Claudin-2 Gene

**DOI:** 10.1371/journal.pone.0085345

**Published:** 2014-03-24

**Authors:** Rana Al-Sadi, Dongmei Ye, Michel Boivin, Shuhong Guo, Mariam Hashimi, Lisa Ereifej, Thomas Y. Ma

**Affiliations:** 1 Department of Internal Medicine, University of New Mexico School of Medicine, Albuquerque, New Mexico, United States of America; 2 Albuquerque Veterans Affairs Medical Center, Albuquerque, New Mexico, United States of America; University of Kansas School of Medicine, United States of America

## Abstract

Defective intestinal epithelial tight junction (TJ) barrier has been shown to be a pathogenic factor in the development of intestinal inflammation. Interleukin-6 (IL-6) is a pleiotropic, pro-inflammatory cytokine which plays an important role in promoting inflammatory response in the gut and in the systemic circulation. Despite its key role in mediating variety inflammatory response, the effect of IL-6 on intestinal epithelial barrier remains unclear. The purpose of this study was to investigate the effect of IL-6 on intestinal epithelial TJ barrier and to delineate the intracellular mechanisms involved using *in-vitro* (filter-grown Caco-2 monolayers) and *in-vivo* model (mouse intestinal perfusion) systems. Our results indicated that IL-6 causes a site-selective increase in Caco-2 intestinal epithelia TJ permeability, causing an increase in flux of small-sized molecules having molecular radius <4 Å. The size-selective increase in Caco-2 TJ permeability was regulated by protein-specific increase in claudin-2 expression. The IL-6 increase in TJ permeability required activation of JNK signaling cascade. The JNK pathway activation of AP-1 resulted in AP-1 binding to its binding sequence on the claudin-2 promoter region, leading to promoter activation and subsequent increase in claudin-2 gene transcription and protein synthesis and TJ permeability. Our in-vivo mouse perfusion showed that IL-6 modulation of mouse intestinal permeability was also mediated by AP-1 dependent increase in claudin-2 expression. In conclusion, our studies show for the first time that the IL-6 modulation of intestinal TJ permeability was regulated by JNK activation of AP-1 and AP-1 activation of claudin-2 gene.

## Introduction

Intestinal mucosal surface is covered by a single layer of columnar epithelial cells. The intestinal epithelial cells have important dual function, absorption of nutrients and fluid needed for the survival of the organism and a barrier function to exclude harmful luminal antigens and microorganisms from intestinal penetration [1], [2]. The intestinal epithelial cells and the tight junctions form the intrinsic intestinal epithelial barrier, serving as a physical and functional barrier against trans-epithelial permeation of luminal substances [1], [2]. The unstirred water layer and the mucus layer coating the epithelial cell surface serve as extrinsic chemical barrier against diffusion of lipophilic molecules, and the lipid bi-layer composition of enterocyte plasma membrane provides a diffusion barrier against trans-membrane permeation of hydrophilic molecules [3]. The intercellular tight junctions act as gate or barrier against paracellular permeation of hydrophilic molecules in-between adjacent cells [1], [3]. It is well-established that in intestinal permeability disorders, the defective intestinal TJ barrier allows paracellular permeation of luminal antigens which can initiate or propagate inflammatory response [1], [4]. Previous studies from our laboratory and others have shown that pro-inflammatory cytokines, including TNF-α, IL-1β, and IFN-γ, cause an increase in intestinal TJ permeability and contribute to the inflammatory process by allowing antigenic penetration [5], [6], [7], [8], [9]. Conversely, anti-inflammatory cytokine IL-10 has been shown to promote intestinal TJ barrier function [10]. In IL-10^−/−^ mice, the development of intestinal inflammation was preceded by an increase in intestinal permeability [10], and enhancement of intestinal TJ barrier with a TJ barrier enhancing agent (AT-1001) prevented the development of intestinal inflammation [11]. Consistent with the pathogenic role of intestinal TJ barrier defect in intestinal inflammation, transgenic mice lacking junctional proteins JAM-A protein or dominant negative-N-cadherin transgenic mice developed spontaneous intestinal inflammation or had worsening intestinal inflammation in response to inflammatory challenges [12], [13]. In clinical studies, a persistent increase in intestinal permeability was predictive of early recurrence of Crohn's disease and poor prognosis, while normalization of intestinal permeability following medical therapy predicted sustained clinical remission [14], [15].

Interleukin-6 is a pro-inflammatory cytokine which is markedly elevated in various inflammatory diseases of the gut, including Crohn's disease, ulcerative colitis, and necrotizing enterocolitis [16], [17]. IL-6 has multiple pro-inflammatory actions and has been shown to play an essential role in the inflammatory process in the gut [17], [18]. A clinical trial of anti-IL-6 receptor monoclonal antibody therapy in patients with active Crohn's disease found that the monoclonal antibody treatment was well-tolerated and produced a significant clinical improvement compared to the placebo treatment [19], [20]. Anti-IL-6 receptor monoclonal antibody has also been shown to be highly effective in the treatment of patients with rheumatoid arthritis [21]. Despite its central importance in inflammatory process, the role of IL-6 in the modulation of intestinal epithelial barrier function remains unclear. Previous studies by Wang et al indicated that IL-6 treatment of Caco-2 BBE monolayers leads to an enhancement of Caco-2 TJ barrier function, as evidenced by a decrease in trans-epithelial flux of paracellular marker dextran (4 kd) [22]. They also showed that intestinal permeability was significantly increased in IL-6^−/−^ mice compared to the WT mice following DSS treatment [22]. The IL-6 treatment also prevented the induced increase in intestinal permeability [23]. In contrast, Tazuke et al and Suzuki et al reported that IL-6 treatment of Caco-2 monolayers produces an increase in TJ permeability [24], [25]. Thus, it remains unclear whether IL-6 has an intestinal barrier protective or disruptive effect.

The major purpose of this study was to clarify the role of IL-6 in the regulation of intestinal epithelial tight junctional barrier, using *in-vitro* (consisting of filter-grown Caco-2 monolayer) and *in-vivo* (recycling mouse intestinal perfusion) model systems. In addition, intracellular signaling pathways and molecular mechanisms that mediate IL-6 effect were also investigated. Our data suggested that IL-6 causes a size-selective increase in intestinal epithelial TJ permeability and that the increase in TJ permeability was regulated by c-jun N-terminal kinase (JNK) pathway activation of claudin-2 gene.

## Materials and Methods

### Ethics statement

The Laboratory Animal Care and Use Committee at the University of New Mexico approved all experimental protocols.

### Cell cultures

Caco-2 cells (passage 19) were purchased from American Type Culture Collection (Rockville, MD), and the cultures were grown in a DMEM with 4.5 g/l glucose, 50 U/ml penicillin, 50 U/ml streptomycin, and 10% FBS. Culture medium was changed every 2 days. Cells were passaged at 70% confluence (typically 5–7 d). Caco-2 cells were plated on Transwell filters and monitored regularly by measuring TER. For experimental purposes, only Caco-2 cells between passages 20 and 26 were used. IL-6 was obtained from R&D systems (Minneapolis, MN). The JNK inhibitor SP-600125 was obtained from Sigma-Aldrich chemicals (St-Louis MO). Unless stated otherwise, IL-6 treatment was given at a dose of 10 ng/ml, SP-600125 at 25 µM.

### Determination of Caco-2 intestinal monolayer resistance and paracellular permeability

The epithelial electrical resistance of the filter-grown Caco-2 intestinal monolayers was measured by using an epithelial volt-ohmmeter (World Precision Instruments, Sarasota, FL) as previously described [5], [26]. For resistance measurements, both apical and basolateral sides of the epithelia were bathed in appropriate buffer solution. Electrical resistance was measured until similar values were recorded on three consecutive measurements. The effect of IL-6 on Caco-2 monolayer paracellular permeability was determined using the paracellular markers urea, mannitol, inulin, and dextran. Known concentrations of permeability marker and its radioactive tracer were added to the apical solution. Low concentrations of permeability markers were used to ensure that negligible osmotic or concentration gradient was introduced. All flux studies were carried out at 37°C. After equilibration for 1 h, basal solution was collected and measured on a Beckman Liquid Scintillation Counter (Beckmann Instruments, Fullerton, CA). All of the electrical resistance and flux experiments were repeated three to six times in triplicates to ensure reproducibility.

### Assessment of TJ protein expression by Western blot analysis

Filter-grown Caco-2 monolayers were treated with IL-6 for varying experimental periods. At the end of the experimental period, Caco-2 monolayers were washed twice with cold PBS, and cells were lysed by using triple detergent lysis buffer. Monolayers were scraped, and the cell lysates were placed in microfuge tubes. Protein concentrations were determined using a Lowry assay. Equal amounts of protein were loaded into microfuge tubes and boiled for 7 min with 2X Laemmli loading buffer (Bio-rad laboratories, Hercules, CA). The lysates were then ran on 4–20% gradient polyacrylamide gels for 2 h. The gels were transferred to nitrocellulose membranes (Bio-rad), blocked with 5% milk and TBS and blotted with appropriate primary and secondary antibodies (Invitrogen, Carlsbad, CA). The membranes were developed using chemiluminescent HRP substrate (Santa Cruz laboratories, Santa Cruz, CA) on Kodak film.

### RNA isolation and reverse transcription

Caco-2 cells (5×10^5^/filter) were seeded into six-well transwell permeable inserts and grown to confluency. Filter-grown Caco-2 cells were then treated with appropriate experimental reagents for desired time periods. At the end of the experimental period, cells were washed twice with ice-cold PBS. Total RNA was isolated using Qiagen RNeasy Kit (Qiagen, ML) according to the manufacturer's protocol. Total RNA concentration was determined by absorbance at 260/280 nm using SpectrraMax 190 (Molecular Devices). The reverse transcription (RT) was carried out using the GeneAmp Gold RNA PCR core kit (Applied Biosystems, Foster city, CA). Two micrograms of total RNA from each sample were reverse transcribed into cDNA in a 40-µl reaction containing 1× RT-PCR buffer, 2.5 mM MgCl_2_, 250 µM of each dNTP, 20 U RNase inhibitor, 10 mM DTT, 1.25 µM random hexamer, and 30 U multiscribe RT. The RT reactions were performed in a thermocycler (PTC-100, MJ Research, Waltham, MA) at 25°C for 10 min, 42°C for 30 min, and 95°C for 5 min.

### Quantification of gene expression using real-time PCR

The real-time PCRs were carried out using ABI prism 7900 sequence detection system and Taqman universal PCR master mix kit (Applied Biosystems, Branchburg, NJ) as previously described [27], [28]. Each real-time PCR reaction contained 10 µl RT reaction mix, 25 µl 2× Taqman universal PCR master mix, 0.2 µM probe, and 0.6 µM primers. Primer and probe design for the real-time PCR was made with Primer Express version 2 from Applied Biosystems. [The primers used in this study are as follows: claudin-2 specific primer pairs consisted of 5′- CTACTGAGAGGTCTGCCAT -3′ (forward), 5′- GGCACCGACATAAGAACTTG -3′ (reverse); probe specific for claudin-2 consisted of FAM 5′- CCTAGGATGTAGCCCACAAGTTGGA -3′ TAMRA; the internal control GAPDH-specific primer pairs consisted of 5′ CCACCCATGGCAAATTCC-3′ (forward), 5′-TGGGATTTCCATTGATGACCAG-3′ (reverse); probe specific for GAPDH consisted of JOE 5′-TGGCACCGTCAAGGCTGAGAACG-3′ TAMRA]. All runs were performed according to the default PCR protocol (50°C for 2 min, 95°C for 10 min, 40 cycles of 95°C for 15 s, and 60°C for 1 min). For each sample, real-time PCR reactions were performed in triplicate, and the average threshold cycle (Ct) was calculated. A standard curve was generated to convert the Ct to copy numbers. Expression of claudin-2 mRNA was normalized with GAPDH mRNA expression. The average copy number of claudin-2 mRNA expression in control samples was set to 1.0. The relative expression of claudin-2 mRNA in treated samples was determined as a fold increase compared with control samples.

### Isolation of nuclear extracts and ELISA-based DNA binding Assay

Filter-grown Caco-2 cells were treated with IL-6 (10 ng/ml) for increasing time points (5–60 min). Cells were washed with ice-cold PBS, scraped, and centrifuged at 14,000 rpm for 30 s. To isolate nuclear proteins, the cell pellets were resuspended in 200 µl hypotonic lysis buffer (Nuclear Extract kit, Active Motif, Carlsbad CA) and incubated on ice for 15 min to lyse the cells. After centrifugation at 14,000 rpm for 30 s, pelleted nuclei were resuspended in 30 µl complete lysis buffer provided from the manufacturer. After incubation on ice for 20 min, the resuspended nuclei were centrifuged at 14,000 rpm for 20 min to collect the nuclear proteins in the supernatant. Nuclear protein concentrations were determined using the Bradford method. The AP-1 DNA-binding activity assay was performed using Trans-AM ELISA-based kits from Active Motif (Carlsbad, CA) according to the manufacturer's protocol. Nuclear extract (5 µg) was added to individual wells on the plate containing the plate-bound oligonucleiotide with consensus AP-1 sequences and incubated for 1 h. Rabbit anti-c-Jun antibody was added to the well to bind c-Jun from the nuclear extract. After incubation for 1 h, anti-rabbit HRP conjugated IgG were added to the well and incubated for 1 h. Subsequently, developing solution was added, and then stop solution was added. The absorbance at 450 nm was determined using the SpectraMax 190 (Molecular Devices,Sunnyvale, CA).

### SiRNA of claudin-2, JNK and AP-1

Targeted siRNAs were obtained from Dharmacon, Inc. (Chicago, IL). Caco-2 monolayers were transiently transfected using DharmaFect transfection reagent (Lafayette, Co). Briefly, 5×10^5^cells/filter were seeded into a twelve-well transwell plate and grown to confluency. Caco-2 monolayers were then washed with PBS twice and 0.5 ml Accell medium was added to the apical compartment of each filter and 1.5 ml were added to the basolateral compartment of each filter. Five nanograms of the siRNA of interest and 2 µl of DharmaFect reagent were added to the apical compartment of each filter. The IL-6 experiments were carried out 96 h after transfection. The efficiency of silencing was confirmed by Western blot analysis.

### Cloning of the claudin-2 promoter region and deletion constructs

The claudin-2 promoter region (Genebank accession no. KF479201) was cloned using GenomeWalker system (Clontech, CA). A 3179-bp DNA fragment (−3322 to −143) was amplified by PCR. The amplification condition was 1 cycle at 94°C for 2 min, followed by 29 cycles at 94°C for 1 min, 50°C for 1 min, and 72°C for 2 min and 1 cycle at 72°C for 5 min. The resultant PCR product was digested with KpnI and Xho Iand inserted into pGL3-basic luciferase reporter vector (Promega). The sequence was confirmed by DNA services at the University of New Mexico. Construction of claudin-2 promoter reporter plasmids was carried out using the pGL-3 basic luciferase reporter vector. Deletions of claudin-2 promoter were done by the PCR method. The PCR conditions were 1 cycle at 94°C for 2 min, followed by 29 cycles at 94°C for 1 min, 50°C for 1 min, and 72°C for 2 min and 1 cycle at 72°C for 5 min. The resultant PCR products were cloned into pGL-3 basic luciferase reporter vector and the sequences were confirmed.

### Transfection of DNA constructs and assessment of promoter activity

DNA constructs of claudin-2 promoter were transiently transfected into Caco-2 cells using transfection reagent lipofectamine 2000 (Life Technologies). Renilla luciferase vector (pRL-TK, Promega) was cotransfected with each plasmid construct as an internal control. Cells (5×10^5^/filter) were seeded into a six-well transwell plate and grown to confluency. Caco-2 monolayers were then washed with PBS twice and 1.0 ml Opti-MEM medium was added to the apical compartment of each filter and 1.5 ml were added to the basolateral compartment of each filter. One microgram of each plasmid construct and 0.25 µg pRL-TK or 2 µl lipofectamine 2000 was preincubated in 250 µl Opti-MEM, respectively. After 5 min of incubation, two solutions were mixed and incubated for another 20 min, and the mixture was added to the apical compartment of each filter. After incubation for 3 h at 37°C, 500 µl DMEM containing 10% FBS were added to both sides of the filter to reach a 2.5% final concentration of FBS. Subsequently, media were replaced with normal Caco-2 growth media 16 h after transfection. Specific experiments were carried out 48 h after transfection. At the completion of specific experimental treatments, Caco-2 cells were washed twice with 1 ml ice-cold PBS, followed by the addition of 400 µl 1× passive lysis buffer, incubated at room temperature for 15 min, scraped and transferred into an Eppendorf tube, and centrifuged for 15 s at 13,000 rpm in a microcentrifuge. Luciferase activity was determined using the dual luciferase assay kit (Promega). Twenty microliters of the supernatant were used for each assay. Luciferase values were determined by Lumat LB 9507 (EG&G Berthold, Oak Ridge, TN). The value of reporter luciferase activities were then divided by that of renilla luciferase activities to normalize for differences in transfection efficiencies. The average activity value of the control samples was set to 1.0. The luciferase activity of claudin-2 promoter in treated samples was determined relative to the control samples.

### Site-directed mutagenesis

Mutagenesis of claudin-2 promoter was performed using the GeneTailor Site-Directed Mutagenesis System (Invitrogen). Briefly, primers (Mutant primer: forward: 5′- TTTCCTTTCTCATGTGTTATTTCTAAAGATAACAAAGACTGAAAGGCATC -3′; Reverse primer: 5′- GATGCCTTTCAGTCTTTGTTATCTTTAGAAATAACACATGAGAAAGGAAA -3′) were generated that included the mutation site flanked by a wild-type sequence on either side. A PCR reaction produced a new complete copy of the plasmid containing the mutation coded for by the primers. The linear PCR product was subsequently transformed into DH5™, which circularized the PCR product and digested any remaining parent plasmid. DNA sequence was then verified by DNA services at the University of New Mexico.

### *In-vivo* Mouse Intestinal Permeability Measurements

The Laboratory Animal Care and Use Committee at the University of New Mexico approved all experimental protocols. The mouse intestinal permeability was measured by recycling small intestinal perfusion as previously described [29], [30], [31]. After the appropriate experimental treatment, mice were anesthetized with isoflurane. After midline incision of the abdomen, 5 cm of intestine segment was isolated and cannulated at the proximal and distal ends with 0.76 mm internal diameter polyethylene tubing. Flushing solution (140 mM NaCl, 10 mM HEPES, pH 7.4) warmed to 37°C was first perfused through the intestine at 1 ml/min for 20 minutes followed by air flush to remove residual contents using a external pump (Bio-Rad Laboratories). This was followed by perfusion of 5 ml perfusate solution (85 mM NaCl, 10 mM HEPES, 20 mM sodium ferrocyanide, 5 mM KCl, 5 mM CaCl_2_, pH 7.4.) containing Texas Red-labeled dextran (10 kDa) in a recirculating manner at 0.75 ml/min for 2 hours. The abdominal cavity was covered with moistened gauze, and body temperature was measured via rectal thermometer, and temperature was maintained at 37.5±0.5°C using a heating lamp. One-milliliter aliquots of test solution were removed at the beginning and end of the perfusion. At the end of the perfusion, the animal was sacrificed and the perfused intestine segment excised and the length measured. The excised intestinal loop was then snap-frozen in optimal cutting temperature compound or used for protein and RNA analysis. Ferrocyanide concentration in the perfusate was measured using colorimetric assay. Texas Red-labeled dextran 10k concentration was measured using an excitation wavelength of 595 nm and an emission wavelength of 615 nm in a microplate reader. Probe clearance was calculated as *C*_probe_ = (*C*_i_ *V*_i_−*C*_f_ *V*_f_)/(*C*_avg_ *TL*). In the equation, *C*_i_ represents the measured initial probe concentration; *C*_f_ represents the measured final probe concentration; *V*_i_ represents the measured initial perfusate volume; *V*_f_ was calculated as *V*_i_([ferrocyanide]_i_/[ferrocyanide]_f_); *C*_avg_ was calculated as (*C*_i_−*C*_f_)/ln(*C*_i_/*C*_f_); *T* represents hours of perfusion; and *L* represents the length of the perfused intestine section in centimeters.

### *Ex-vivo* intestinal tissue epithelial resistance measurement in Ussing Chambers

The small intestinal tissue epithelial resistance was measured using Ussing chamber 24 h after i.p. injection of IL-6. After midline incision of the abdomen, small intestine was dissected out and 1–1.5 cm sample was vertically mounted in Ussing chambers that provided an exposed area of 0.126 cm^2^ as described previously with modifications. The tissues were bathed with Krebs-Ringer bicarbonate (KRB) solution (128 mM NaCl, 5.1 mM KCl, 1.4 mM CaCl_2_, 1.3 mM MgCl_2_, 21 mM NaHCO_3_, 1.3 mM KH_2_PO_4_, 10 mM NaH_2_PO_4_, pH 7.4). Solutions were gassed with 95% O_2_/5% CO_2_. After a 15 min equilibration period, the transepithelial electrical voltage and current was measured at 5 min intervals until 30 min using EVC 4000 Precision V/I clamp device (World Precision Instruments, Sarasota, USA). Intestinal tissue resistance was calculated using Ohm's law(R = V/I). The experiment was repeated a minimum of three times using different tissue sample each time.

### Animal surgery and *in-vivo* transfection of claudin-2 and AP-1 siRNA

Mice were fasted for 24 hours prior to the surgery. Mice were anesthetized with isoflurane (4% for surgical induction, 1% for maintenance) using oxygen as carrier during surgical procedures. Surgical procedures were performed using sterile technique. The abdomen was opened by a midline incision, and a 6 cm of intestine segment was isolated at the proximal and distal ends and tied with sutures. 0.5 ml of siRNA transfection solution (containing Accell medium; 2.5 nmol claudin-2 or AP-1 siRNA and 50 µl transfecting agent lipofectamine) was introduced into the isolated intestine segment (surface area 6 cm^2^) for 1 hr transfection period. Control animals underwent sham-operation, where the siRNA transfection solution contained Accell medium; 2.5 nmol non-target siRNA and 50 µl transfecting agent lipofectamine. The abdominal cavity was covered with moistened gauze. Body temperature was monitored continuously with a rectal probe and maintained at 37.5±0.5°C using a heating pad. After 1 hour transfection period, each end of the intestinal segment was untied and the intestine placed back in the abdominal cavity, and the abdomen was closed. Three days following transfection, functional studies of intestinal epithelial barrier were performed. The surgery and the *in-vivo* transfection procedures had no effect on the food intake and the body weight of the animals during the experimental period. The average animal weight averaged between 23 to 25 g during the experimental period.

### Statistical analysis

Statistical significance of differences between mean values was assessed with Student's *t*-tests for unpaired data and ANOVA analysis whenever required. All reported significance levels represent two-tailed *P* values. A *P* value of <0.05 was used to indicate statistical significance. All experiments were repeated at least three times to ensure reproducibility.

## Results

### IL-6 modulation of Caco-2 intestinal epithelial tight junction barrier

**Figure 1:**
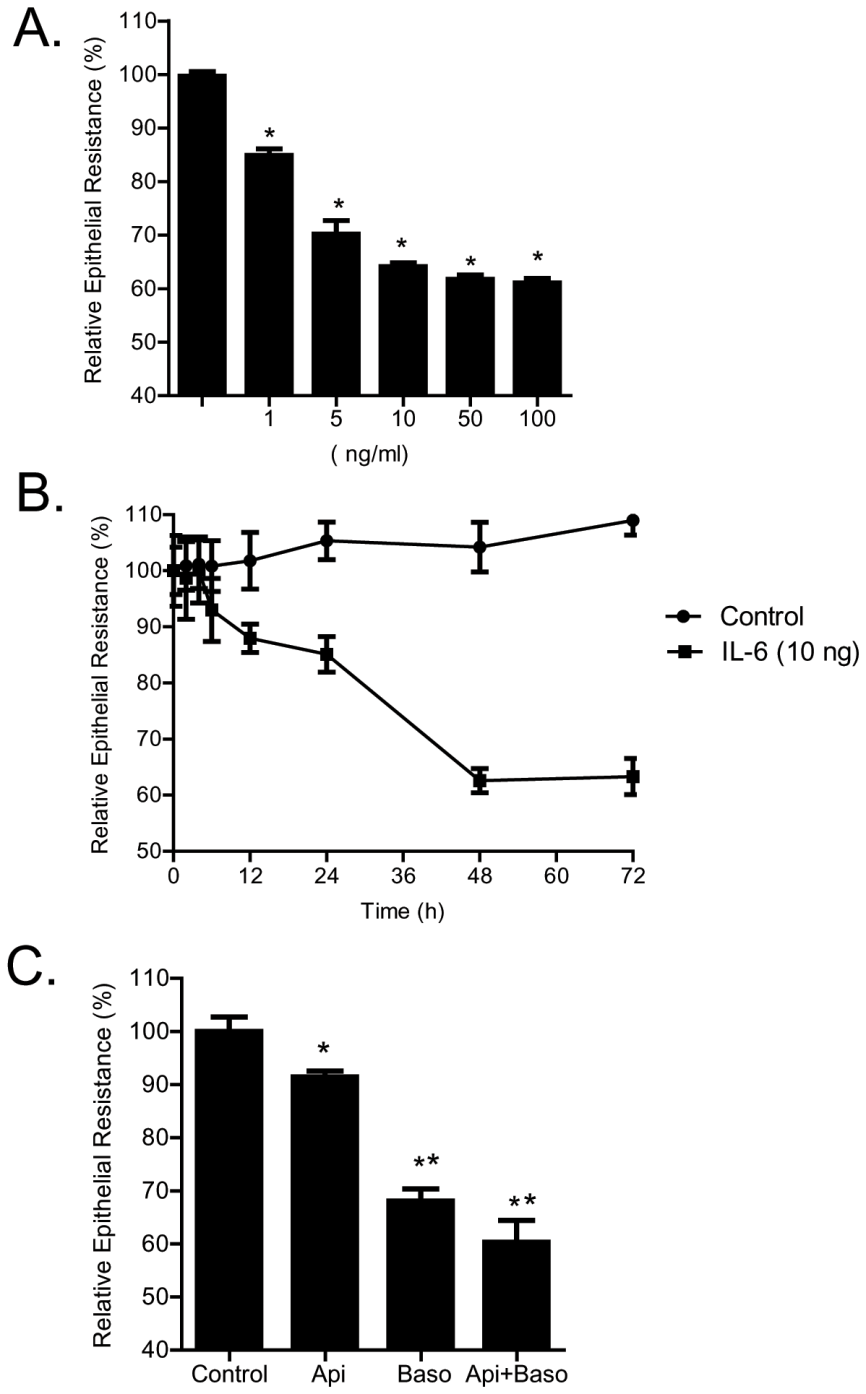
Effect of IL-6 on trans-epithelial electrical resistance (TER) in Caco-2 intestinal epithelial monolayers. (A) IL-6 caused a dose-dependent decrease in TER over 48-h experimental period (*n* = 4). *p<0.001 vs. control. (B) IL-6 (10 ng/ml) caused a time-dependant decrease in Caco-2 TER (*n* = 4). (C) Membrane specificity of IL-6 effect on Caco-2 epithelial resistance. IL-6 (10 ng/ml) was added to either apical, basolateral, or combined apical and basolateral compartments (*n* = 4). _*_, *p*<0.05 vs. control; _**_, *p*<001 vs. IL-6 addition to apical compartment alone.

The dose- and time-course of IL-6 effect on Caco-2 TJ barrier function was assessed by measuring Caco-2 TER. IL-6 caused a dose-dependent drop in Caco-2 TER (Fig. 1A) during the 48-hr experimental period; IL-6 concentration of 10 ng/ml caused a maximal drop in TER (p<0.001) and increasing the IL-6 concentration above 10 ng/ml did not cause additional drop in TER. (In all subsequent studies, unless otherwise indicated, a dose of 10 ng/ml of IL-6 was used). IL-6 (10 ng/ml) also caused a time-dependent drop in Caco-2 TER, and by 48 h there was about a 40% drop in TER (Fig. 1B). The membrane specificity of IL-6 (10 ng/ml) effect on Caco-2 TER was also examined (Figure 1C). IL-6 was added to either the apical, basolateral or combined apical and basolateral compartments. The addition of IL-6 to the apical compartment caused a small but significant decrease in TER (*p*<0.05). The addition of IL-6 to the basolateral compartment caused a greater drop in TER than apical compartment alone. The addition of IL-6 to both apical and basolateral compartments caused a similar drop in TER as the basolateral addition alone. Although there appeared to be a trend toward a greater drop in TER, this was not statistically significant. (In all subsequent experiments, IL-6 was added only to the basolateral compartment).

**Figure 2:**
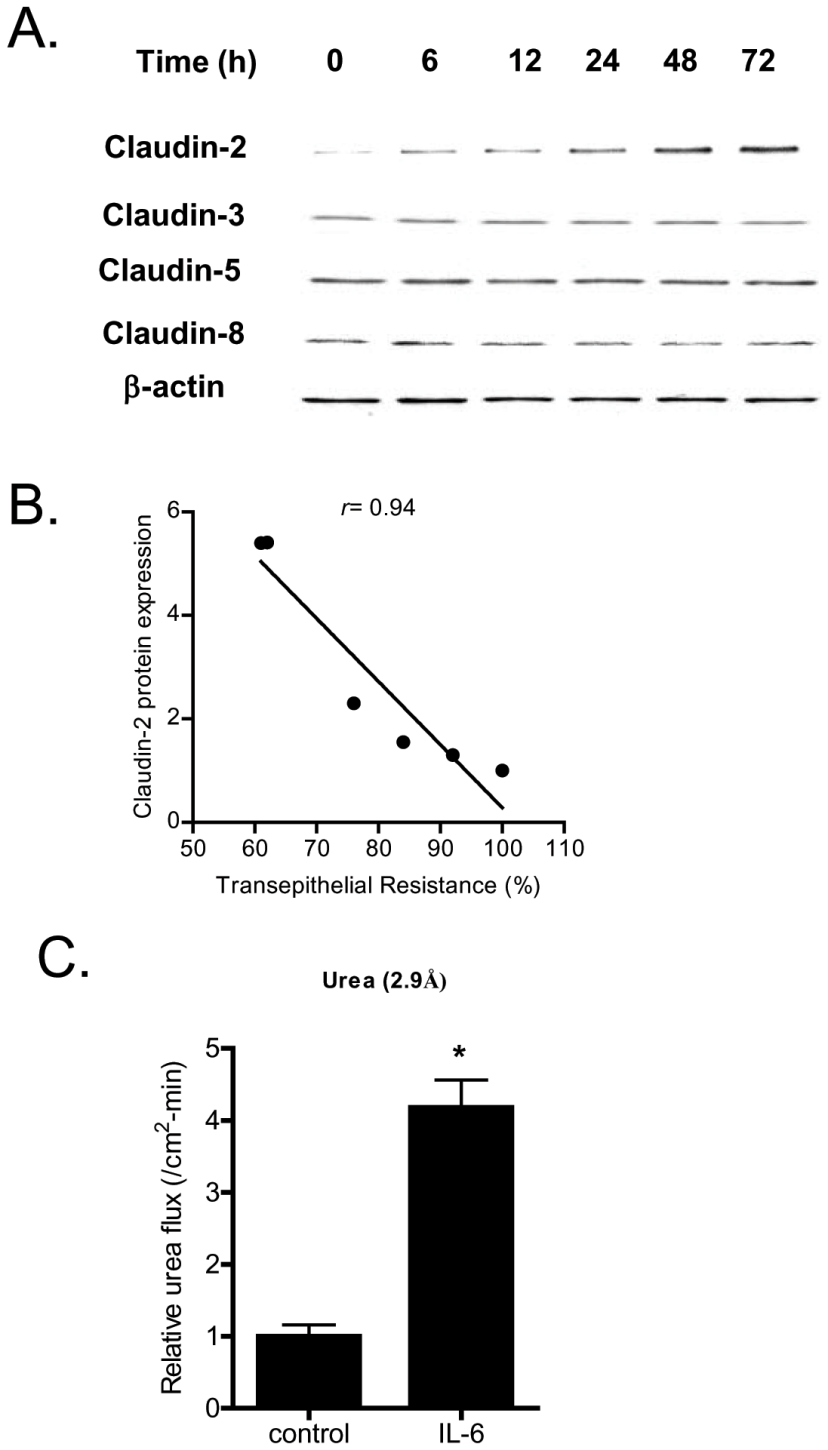
Effect of IL-6 on Caco-2 tight junction proteins expression. (A) IL-6 caused a time-dependent increase in claudin-2 protein expression but not claudins 3, 5, and 8 as assessed by Western Blot analysis. (B) Graph of claudin-2 protein expression vs. relative decrease in transepithelial resistance following IL-6 treatment (relative correlation coefficient, *r* = 0.94). (C) IL-6 treatment (10 ng/ml) of Caco-2 monolayers over 48 h experimental period induced a significant increase in mucosal-to-serosal urea flux (*n* = 4). _*_, *p*<0.001 vs. control.

Next, the time-course of IL-6 effect on expression of transmembrane TJ proteins, claudin-2, claudin-3, claudin-5, claudin-8, and occludin, and cytoplasmic plaque protein, ZO-1, was determined. IL-6 caused a time-dependent increase in claudin-2 protein expression (Fig. 2A) but did not affect the expression of other transmembrane proteins or ZO-1 (data not shown). The time-course of IL-6 induced increase in claudin-2 expression correlated linearly with the drop in Caco-2 TER with a relative correlation coefficient of r = 0.96 (Fig. 2CB), suggesting the possibility that the increase in claudin-2 expression may be responsible for the drop in Caco-2 TER.

**Figure 3:**
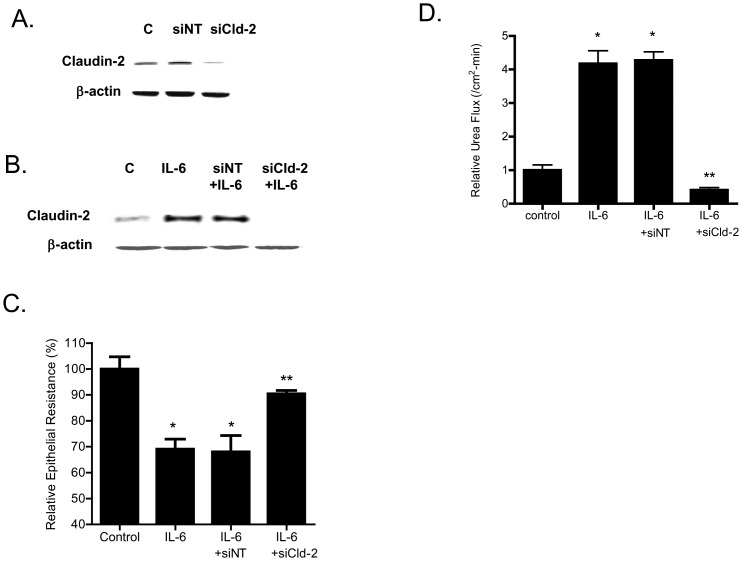
Effect of siRNA- induced knock-down of claudin-2 on Caco-2 intestinal epithelial permeability. (A) SiRNA claudin-2 transfection resulted in a near-complete depletion of claudin-2 expression as assessed by Western blot analysis (4 days post-transfection). (B) IL-6 caused an increase in claudin-2 protein expression. Knocking-down claudin-2 by siRNA transfection prevented the IL-6 induced increase in claudin-2 expression as assessed by Western Blot analysis. (C) siRNA induced knock down of claudin-2 prevented the IL-6 induced drop in Caco-2 TER. (*n* = 6). _*_, *p*<0.001 vs. control; _**_, *p*<001 vs. IL-6 treatment. (D) Claudin-2 depletion prevented the IL-6 induced increase in urea flux across Caco-2 monolayers. (*n* = 6). _*_, *p*<0.001 vs. control; _**_, *p*<001 vs. IL-6 treatment.

In the following studies, the effect of IL-6 on mucosal-to-serosal flux of increasing-sized paracellular markers, urea (mol wt = 60 g/mol) (Fig. 2C), mannitol (mol wt = 182 g/mol), inulin (mol wt = 5,000 g/mol), and dextran (mol wt = 10,000 g/mol), was examined in filter-grown Caco-2 monolayers (data not shown). IL-6 caused about a 3- to 4-fold increase in trans-epithelial flux of smaller-sized paracellular marker urea (molecular radius of 2.9 Å), but did not affect the flux of larger-sized molecules including mannitol (molecular radius of 4.1 Å), inulin (molecular radius of 15 Å) or dextran-10kd (molecular radius of 23.6 Å) (data not shown) [32]. These results suggested that the IL-6 effect on Caco-2 TJ permeability was size-dependent and limited to smaller-sized molecules. Since claudin-2 dependent pore pathways have been shown to be “perm selective” allowing paracellular flux of ions and smaller sized molecules <4.0 Å in molecular radius [32], [33], our results above also suggested the possibility that the IL-6 induced drop in Caco-2 TER and the size-selective increase in urea flux was due to an increase in claudin-2 dependent pore pathways [33]. To investigate this possibility, Caco-2, claudin-2 expression was selectively silenced by claudin-2 siRNA transfection. As shown in Fig. 3A, claudin-2 siRNA transfection caused a near-complete knock-down of claudin-2 in filter-grown Caco-2 monolayers; and claudin-2 siRNA transfection inhibited the IL-6 induced increase in claudin-2 expression (Fig. 3B). The siRNA induced knock-down of claudin-2 inhibited the IL-6 induced drop in Caco-2 TER (Fig. 3C). The transfection of Caco-2 monolayers with a negative control (scrambled) siRNA did not prevent the increase in claudin-2 expression or the drop in TER (Fig. 3B, 3C). The claudin-2 siRNA transfection also inhibited the IL-6 induced increase in urea flux (Fig. 3D), confirming that the increase in claudin-2 was necessary for the size-selective increase in Caco-2 TJ permeability.

### IL-6 modulation of Caco-2 TJ barrier is regulated by JNK signaling pathway

**Figure 4:**
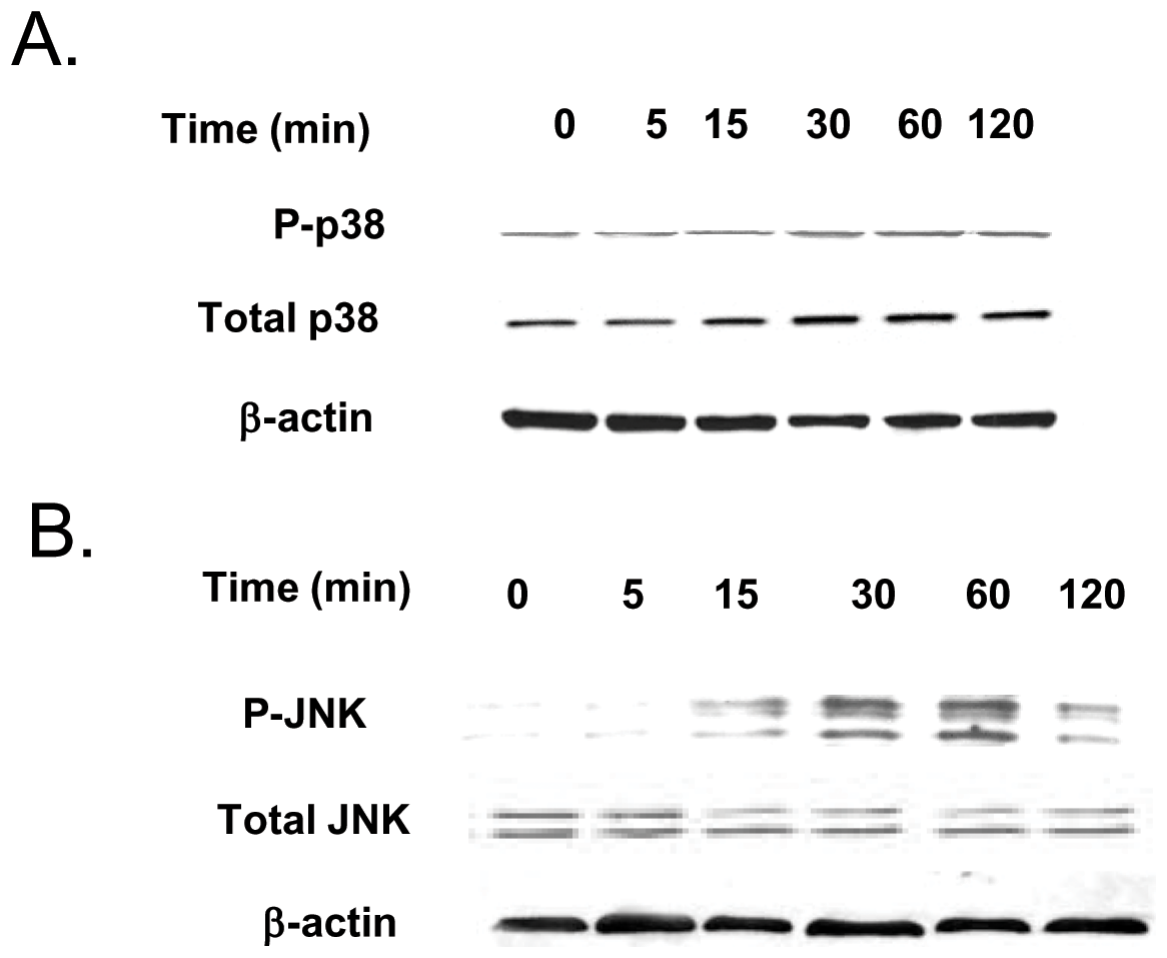
Effect of IL-6 on activation of the p38 kinase and JNK phosphorylation. (A) IL-6 treatment of Caco-2 monolayers did not affect the phosphorylation of p38 kinase as assessed by Western Blot of phosph-p38 kinase. (B) IL-6 treatment caused a time-dependent increase in JNK phosphorylation in Caco-2 monolayers.

**Figure 5:**
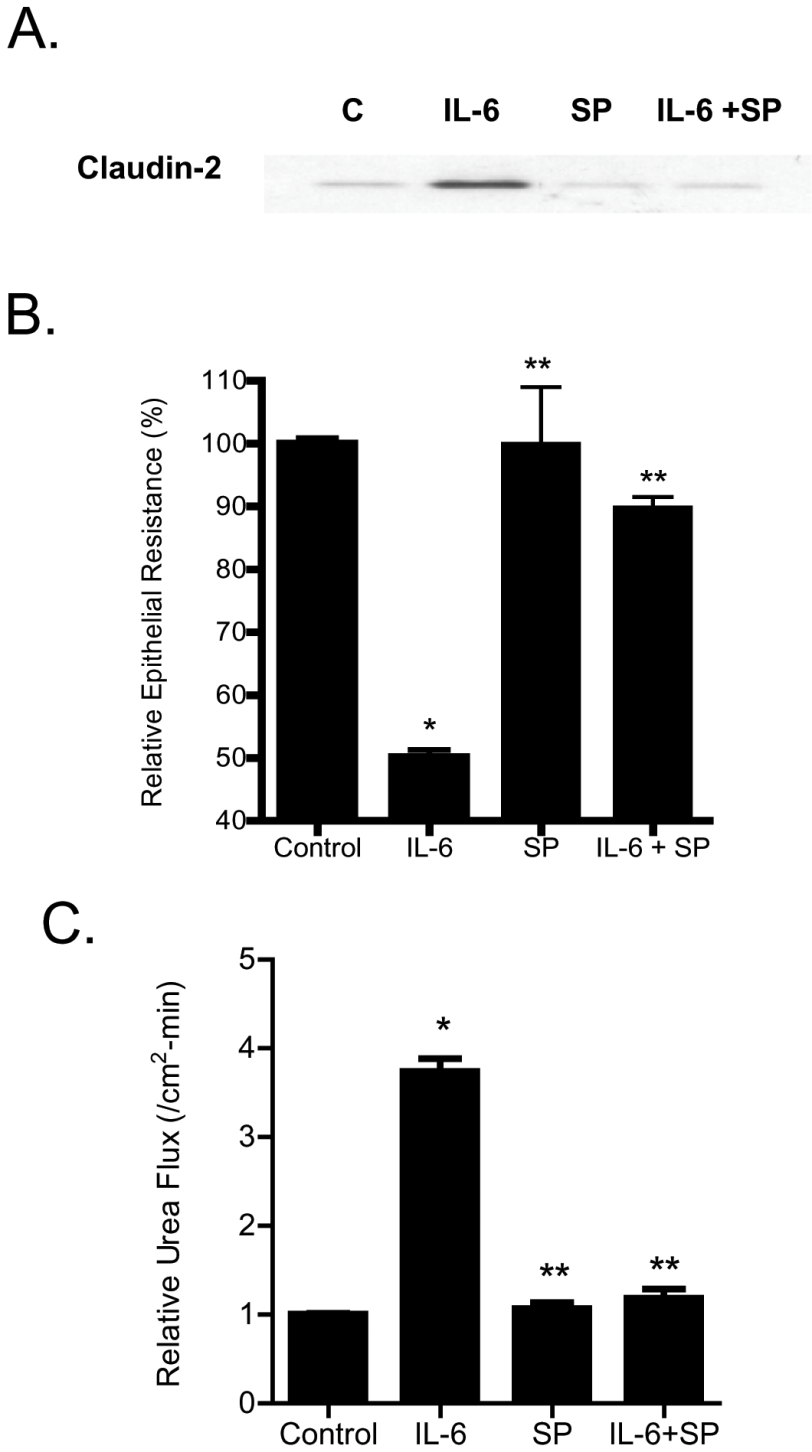
Effect of inhibition of JNK pathway on IL-6 induced increase in Caco-2 TJ permeability. (A) Pre-treatment with JNK inhibitor SP-600125 (25 µM) prevented the IL-6 induced increase in claudin-2 protein expression. (B) SP-600125 pre-treatment prevented the IL-6 induced drop in Caco-2 TER (*n* = 6). _*_, *p*<0.001 vs. control; _**_, *p*<001 vs. IL-6 treatment. (C) SP-600125 pre-treatment prevented the IL-6 induced increase in urea flux in filter-grown Caco-2 monolayers. (*n* = 6). _*_, *p*<0.001 vs. control; _**_, *p*<001 vs. IL-6 treatment.

**Figure 6:**
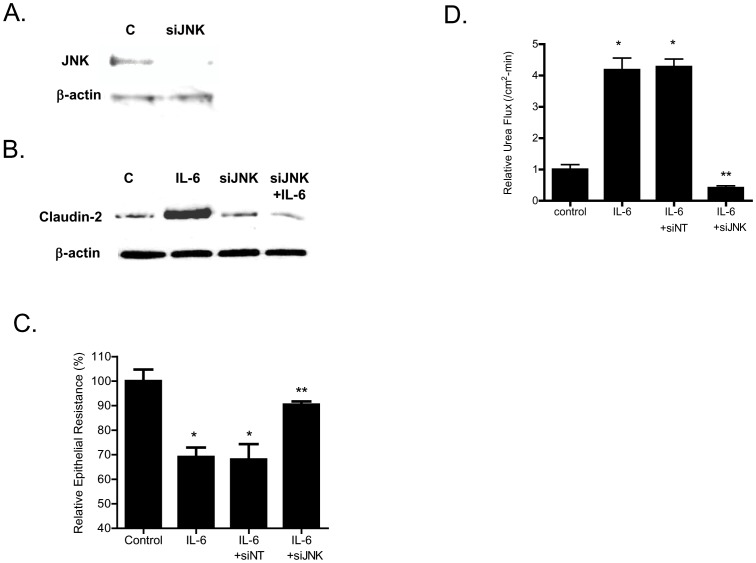
Effect of siRNA- induced knock-down of JNK on Caco-2 intestinal epithelial permeability. (A) JNK siRNA transfection resulted in a near-complete depletion of JNK expression. (B) SiRNA-induced knock down of JNK prevented the IL-6 induced increase in claudin-2 expression. (C) SiRNA induced knock down of JNK abolished the IL-6 induced drop in Caco-2 TER (*n* = 4). _*_, *p*<0.001 vs. control; _**_, *p*<001 vs. IL-6 treatment. (D) JNK siRNA transfection prevented the IL-6 increase in urea flux in Caco-2 monolayers (*n* = 4). _*_, *p*<0.001 vs. control; _**_, *p*<001 vs. IL-6 treatment.

In the following studies, the intracellular signaling pathways responsible for the IL-6 effect on Caco-2 TJ barrier were examined. Previous studies in different cell types indicated that IL-6 causes an activation of MAP kinases p38 kinase and c-Jun N-terminal kinase (JNK) [34], [35]. The IL-6 effect on Caco-2 MAP kinase activation was assessed by measuring the phophorylation of p38 kinase or JNK. The IL-6 treatment of filter-grown Caco-2 monolayers did not affect p38 kinase phosphorylation but caused an increase in JNK phosphorylation (Fig. 4A, 4B), suggesting that IL-6 causes activation of JNK but not p38 kinase in Caco-2 cells. Next, the possible involvement of JNK signaling pathway in IL-6 regulation of claudin-2 expression and Caco-2 TJ barrier was examined. In these studies, IL-6 induced activation of JNK was inhibited by JNK inhibitor SP600125. The pre-treatment of filter-grown Caco-2 monolayers with SP600125 (25 µM) prevented the IL-6 induced increase in claudin-2 expression (Fig. 5A). The SP600125 pre-treatment also resulted in inhibition of IL-6 induced drop in Caco-2 TER (Fig. 5B) and increase in urea flux (Fig. 5C), suggesting that JNK activation was required for the IL-6 increase in Caco-2 TJ permeability. To further validate the involvement of JNK in IL-6 effect, JNK expression was silenced in Caco-2 monolayers by JNK siRNA transfection (Fig. 6A). The JNK siRNA transfection inhibited the IL-6 induced increase in claudin-2 expression (Fig. 6B). The siRNA induced knock-down of JNK also inhibited the IL-6 induced drop in Caco-2 TER (Fig. 6C) and increase in urea flux (Fig. 6D). The transfection with negative control siRNA (scramble) did not affect the drop in Caco-2 TER or the increase in urea flux. Together, these data suggested that the IL-6 induced increase in claudin-2 expression and Caco-2 TJ permeability was regulated by JNK signaling pathway.

### Mechanism of IL-6 regulation of claudin-2 gene

**Figure 7:**
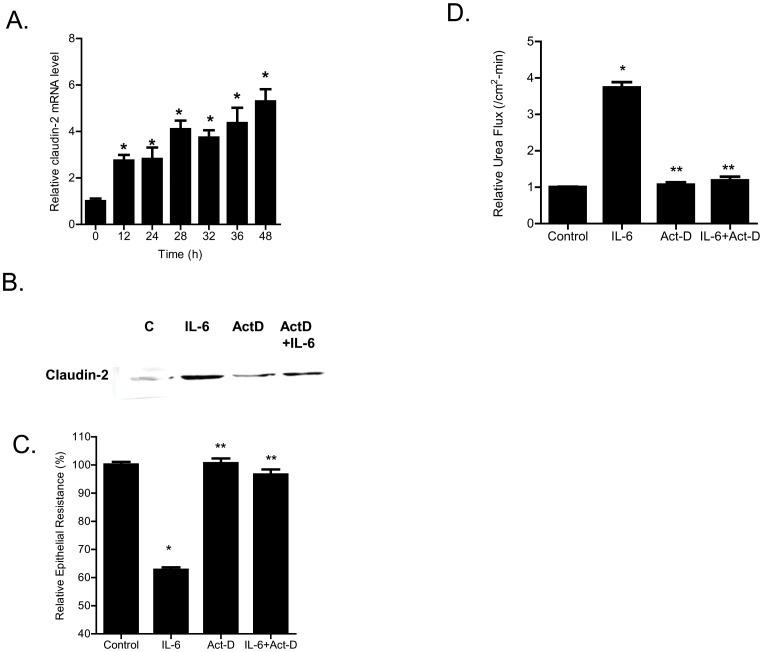
Effect of mRNA transcription inhibitor on IL-6 induced increase in Caco-2 TJ permeability. (A) IL-6 caused a time-dependent increase in claudin-2 mRNA expression as assessed by real-time PCR (*n* = 8). _*_, *p*<0.001 vs. control. (B) mRNA polymerase inhibitor actinomycin-D (100 µg/ml) prevented the IL-6 induced increase in claudin-2 protein expression as assessed by Western Blot analysis. (C) Pre-treatment with actinomycin-D prevented the IL-6 induced drop in Caco-2 TER (*n* = 4). _*_, *p*<0.001 vs. control; _**_, *p*<0.001 vs. IL-6 treatment. (D) Pre-treatment with actinomycin-D also prevented the IL-6 induced increase in urea flux in Caco-2 monolayers (*n* = 4). _*_, *p*<0.001 vs. control; _**_, *p*<0.001 vs. IL-6 treatment.

**Figure 8:**
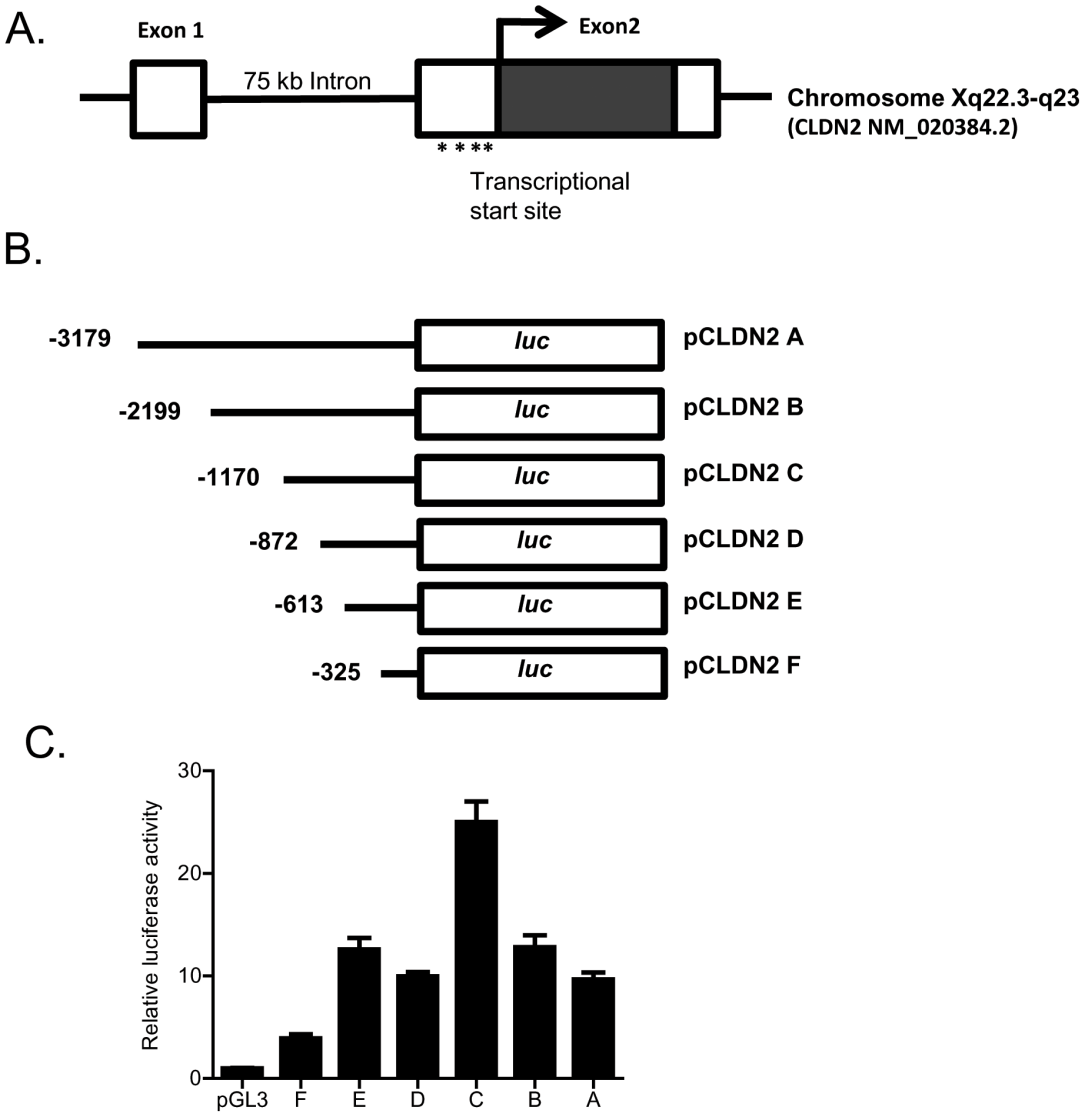
Determination of claudin-2 minimal promoter region. (A) Schematic diagram showing claudin-2 gene mapped to chromosome Xq22.3-q23 region, and organized into 2 exons. The transcription and translation start sites were located in exon 2. The exon 1 and exon 2 were separated by 7.5 kb intron. The 5′ untranslated region was located in exon 1. (B) Schematic diagram showing the deletion constructs of claudin-2 gene that were generated. (C) Transfection of FL and deletion constructs of claudin-2 promoter in Caco-2 monolayers. Transfection of full length (pCLDN2 A) promoter region caused a 10-fold increase in the luciferase activity compared to the pGL3 basic vector alone when transfected into the Caco-2 cells (*n* = 8). The deletion construct −1170 (pCLDN2 C) exhibited the maximal promoter activity (*n* = 8). _*_, *p*<0.05 vs. pGL3; _**_, *p*<0.001 vs. full length promoter region.

In the following studies, the down-stream mechanisms and the molecular processes involved in IL-6 modulation of claudin-2 protein expression was examined. The IL-6 effect on Caco-2 claudin-2 mRNA was examined using real-time PCR. IL-6 caused a time-dependent increase in claudin-2 mRNA expression (Fig. 7A), suggesting the possibility that the increase in claudin-2 protein level may be due to the increase in mRNA transcription. The treatment with a potent transcription inhibitor actinomycin-d (100 µg/ml), at a dose that inhibits Caco-2 transcription activity [36], prevented the IL-6 induced increase in claudin-2 expression (Fig. 7B). Actinomycin-d also inhibited the IL-6 induced drop in Caco-2 TER (Fig. 7C) and increase in urea flux (Fig. 7D), suggesting that the increases in claudin-2 expression and TJ permeability were dependent on claudin-2 mRNA transcription. In the following series of studies, the IL-6 effect on claudin gene activity was examined. To determine the genomic structure of claudin-2, the human genome database was searched with claudin-2 cDNA (Genbank accession No NM 020384). BLAST analysis showed that claudin-2 gene was mapped to chromosome Xq22.3-q23 region, and organized into 2 exons. The transcription and translation start sites were located in exon 2. The exon 1 and exon 2 were separated by 7.5 kb intron. The 5′ untranslated region was located in exon 1 (Fig. 8A). The transcriptional start site of claudin-2 was determined by 5′RACE and a 3,179 bp claudin-2 promoter region up-stream of transcriptional start site was cloned into a pGL3 basic vector (having luciferase as the reporter gene). The sequence of the cloned claudin-2 promoter region is shown in Fig. S1. [The promoter sequence was deposited in Genbank with accession number KF479201.]

To delineate the minimal promoter region having the maximal transcriptional activity, a progressively increasing 5′ deletion constructs were generated (total of 6) and subcloned into the pGL3 basic vector (Fig. 8B). The deletion constructs were then transfected into 90% confluent Caco-2 cells and the promoter activity determined by luciferase assay (Fig. 8C). As shown in Fig. 8C, the full-length (FL) 3,179 bp promoter construct (pCLDN2 A) had a baseline promoter activity ∼10 fold higher than the PGL3 basic control. Progressive deletion extending up to 2 kb from the 5′ end of the FL promoter (pCLDN2 C) resulted in an increase in promoter activity compared to the FL promoter. Further deletion of 5′ end resulted in a progressive decrease in promoter activity (Fig. 8C). There was a large drop in promoter activity between DNA constructs −1170 (pCLDN2 C) and −862 (pCLDN2 D) and between constructs −613 (pCLDN2 E) and −326 (pCLDN2 F), suggesting a presence of positive regulatory elements in these regions. Based on these results, we identified −1170 to 0 (pCLDN2 C) as a minimal claudin-2 promoter region having the maximal promoter activity.

**Figure 9:**
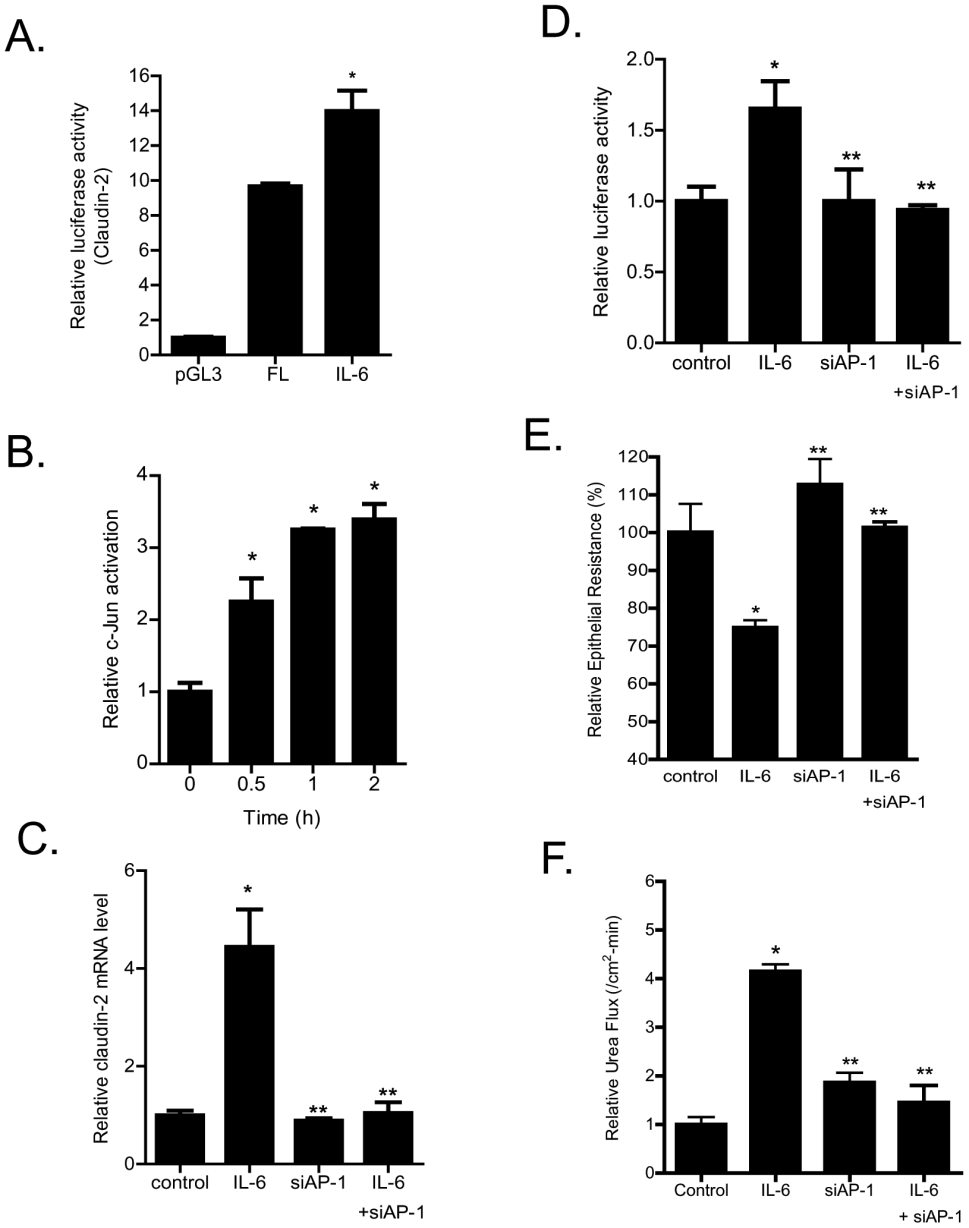
Effect of siRNA induced AP-1 knock-down on IL-6 increase in claudin-2 promoter activity and Caco-2 TJ permeability. (A) IL-6 caused a significant increase in claudin-2 promoter activity as assessed by luciferase assay (*n* = 8). _*_, *p*<0.001 vs. control. (B) IL-6 caused a time-dependent increase in c-JUN DNA binding to AP-1 standard DNA site as assayed by ELISA-based DNA binding assay (*n* = 6). _*_, *p*<0.001 vs. control. (C) siRNA induced knock-down of AP-1 prevented the IL-6 increase in claudin-2 mRNA expression (*n* = 8). _*_, *p*<0.0001 vs. control; _**_, *p*<0.0001 vs. IL-6 treatment. (D) AP-1 depletion by siRNA transfection prevented the IL-6 induced increase in claudin-2 promoter activity (*n* = 4). _*_, *p*<0.001 vs. control; _**_, *p*<0.001 vs. IL-6 treatment. (E) siRNA induced knock down of AP-1 abolished the IL-6 induced drop in Caco-2 TER (*n* = 4). _*_, *p*<0.001 vs. control; _**_, *p*<001 vs. IL-6 treatment. (F) Knocking-down AP-1 by siRNA transfection prevented the IL-6 increase in urea flux in Caco-2 monolayers (*n* = 4). _*_, *p*<0.001 vs. control; _**_, *p*<001 vs. IL-6 treatment.

Next, the effect of IL-6 on claudin-2 promoter activity was determined. The IL-6 treatment of Caco-2 cells transfected with plasmid vector encoding the FL claudin-2 promoter region resulted in a significant increase in promoter activity (Fig. 9A). In the following studies, the transcription factor and the molecular determinants that regulate the IL-6 effect on claudin-2 promoter activity and Caco-2 TJ permeability were determined. We examined the possibility that JNK activated AP-1 plays a role in IL-6 regulation of claudin-2 promoter activity and Caco-2 TJ permeability. [C-Jun is a direct enzymatic substrate of JNK, and c-Jun in combination with c-Fos form the heterodimeric transcription factor AP-1 [37], [38].] The IL-6 effect on AP-1 activation was determined in filter-grown Caco-2 monolayers using an ELISA based DNA binding assay. IL-6 caused a rapid activation of AP-1 as evidenced by an increase in AP-1 binding to the DNA probe (Fig. 9B). To confirm the role of AP-1 in regulating claudin-2 gene activity, AP-1 expression was selectively knock-down by siRNA transfection. The siRNA induced knock-down of AP-1inhibited the IL-6 induced increase in claudin-2 mRNA expression (Fig. 9C) and promoter activity (Fig. 9D). AP-1 knock-down also inhibited the IL-6 induced drop in Caco-2 TER and increase in urea flux (Fig. 9E, 9F). These data suggested that JNK activated AP-1 plays an important regulatory role in claudin-2 gene activation and TJ barrier regulation.

**Figure 10:**
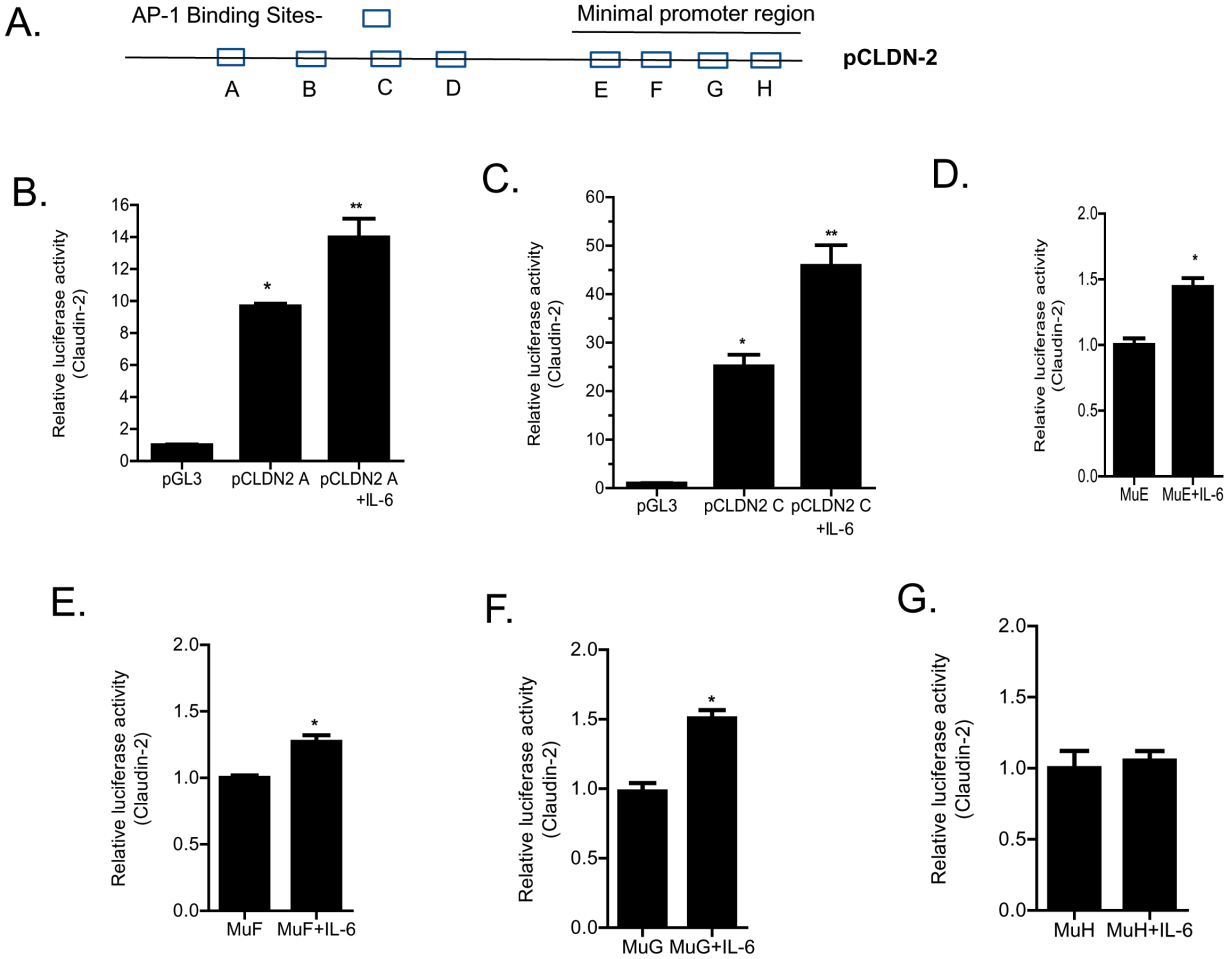
Identification of DNA binding sequence responsible for IL-6 induced increase in claudin-2 promoter activity. (A) Schematic diagram showing 8 potential AP-1 binding sites within the claudin-2 promoter region. Four binding sites were located outside the minimal promoter region (trans-AP-1) and four sites were within the minimal promoter region (cis-AP-1). (B) IL-6 treatment caused an increase in promoter activity in the FL promoter construct (pCLDN2 A) (*n* = 6). _*_, *p*<0.001 vs. control; _**_, *p*<001 vs. IL-6 treatment. (C) IL-6 caused a similar increase in promoter activity in the deletion construct lacking the trans-AP-1 sites (pCLDN2 C) (*n* = 6). _*_, *p*<0.001 vs. control; _**_, *p*<001 vs. IL-6 treatment. The site-directed mutagenesis of three up-stream cis-AP-1 binding sequences (E, F, G) did not affect the IL-6 induced increase in claudin-2 promoter activity (D), (E), (F) (*n* = 6). _*_, *p*<0.001 vs. control; _**_, *p*<001 vs. IL-6 treatment. The mutation of the down-stream cis-AP-1 binding (sequence H) prevented the IL-6 induced increase in claudin-2 promoter activity (*n* = 6) (G).

The molecular determinants on claudin-2 promoter responsible for the IL-6 induced increase in claudin-2 promoter activity were also determined. Based on the above studies showing the requirement of AP-1 in claudin-2 promoter activity, potential AP-1 binding sites on claudin-2 promoter were determined. Using Genomatix software, we have identified 8 potential AP-1 binding sites within the FL claudin-2 promoter region. Four binding sites were located outside the minimal promoter region (pCLDN2C) (trans-AP-1) and four sites were within the minimal promoter region (cis-AP-1) (Fig. 10A). To determine whether the trans-AP-1 sites were involved in the IL-6 effect on promoter activity, the IL-6 effect on deletion construct lacking the trans-AP-1 sites (pCLDN2C) was examined. As shown in Fig. 10B, IL-6 caused an increase in promoter activity in the FL promoter construct (pCLDN2A) compared to the control (*p*<0.05). IL-6 caused a similar increase in promoter activity in the deletion construct lacking the four trans-AP-1 sites (pCLDN2 C) (Fig. 10C), suggesting that the trans-AP-1 sites were not required for the IL-6 induced up-regulation of claudin-2 promoter activity. To determine the specific cis-AP-1 binding site responsible for the up-regulation of the promoter activity, each of the four cis-AP-1 motifs was mutated in the DNA construct pCLDN2 C, which encodes the minimal promoter region (−1170) (Fig. 10A). The mutation of three up-stream cis-AP-1 binding sequences (E, F, G) did not affect the IL-6 induced increase in promoter activity (Fig. 10D, 10E, 10F); however, the mutation of the down-stream cis-AP-1 binding sequence H (CTGCCAACCTAA) completely inhibited the IL-6 induced increase in promoter activity (Fig. 10G). These results suggested that the down-stream AP-1 binding motif H was the molecular determinant mediating the IL-6 induced increase in promoter activity.

### IL-6 effect on mouse intestinal permeability in-vivo

**Figure 11:**
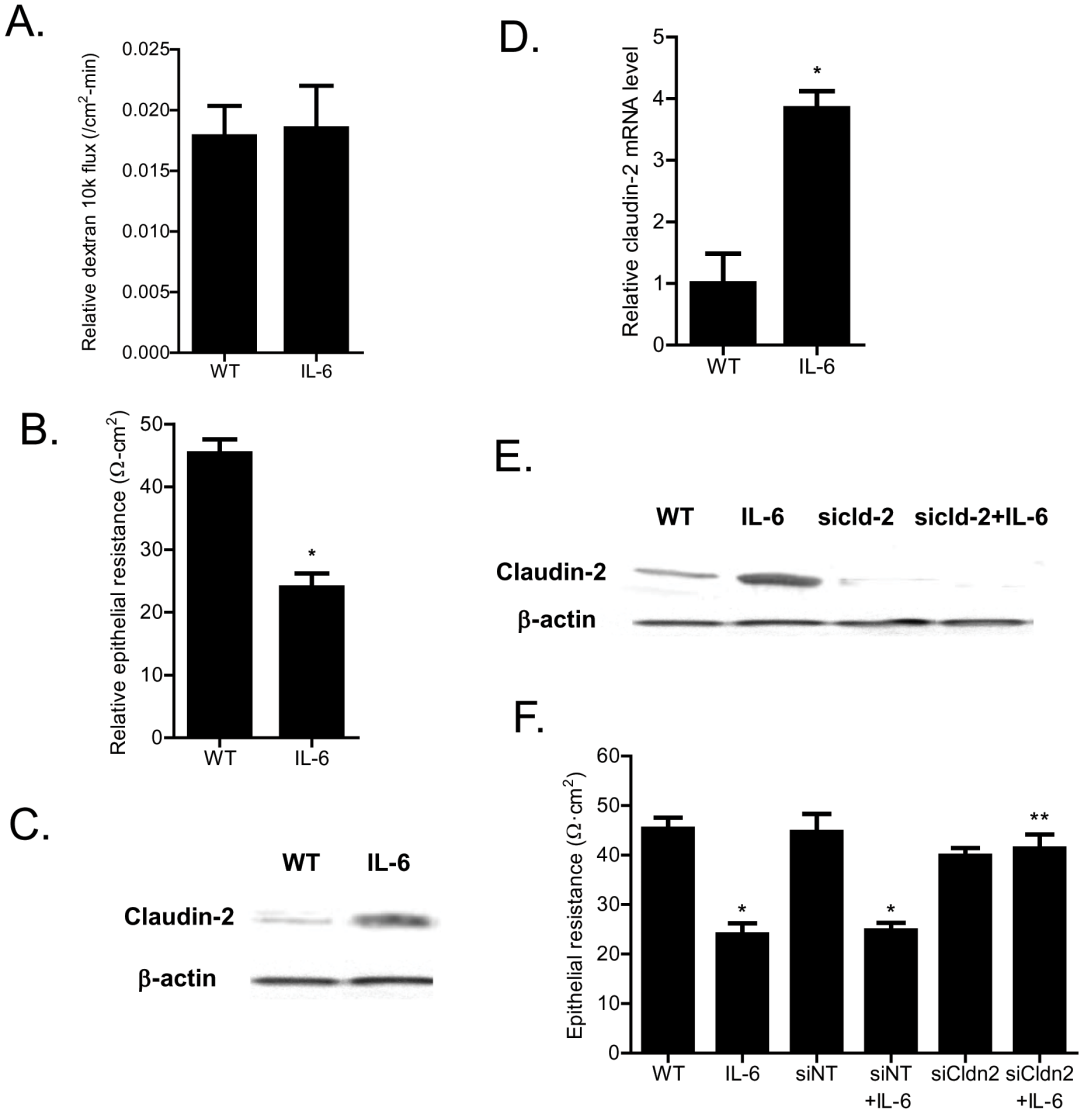
Effect of IL-6 on mouse small intestinal permeability and claudin-2 expression *in-vivo*. (A) Intraperitoneal treatment of IL-6 (5 µg) did not affect the mucosal-to-serosal flux of dextran 10K in mouse intestinal tissues (n = 5). (B) IL-6 treatment caused a drop in mouse intestinal TER as measured in Ussing chambers. _*_, *p*<0.001 vs. control. (C) IL-6 caused an increase in mouse intestinal tissue claudin-2 expression as assessed by Western Blot analysis. (D) IL-6 treatment resulted in an increase in claudin-2 mRNA levels in mouse intestinal tissue (*n* = 5). _*_, *p*<0.0001 vs. control. (E) Claudin-2 siRNA transfection *in-vivo* prevented the IL-6 induced increase in mouse intestinal claudin-2 protein expression as assessed by Western blot analysis. (F) Claudin-2 siRNA transfection *in-vivo* prevented the IL-6 induced drop in mouse intestinal TER as measured in Ussing chambers (*n* = 5). _*_, *p*<0.001 vs. control; _**_, *p*<001 vs. IL-6 treatment.

**Figure 12:**
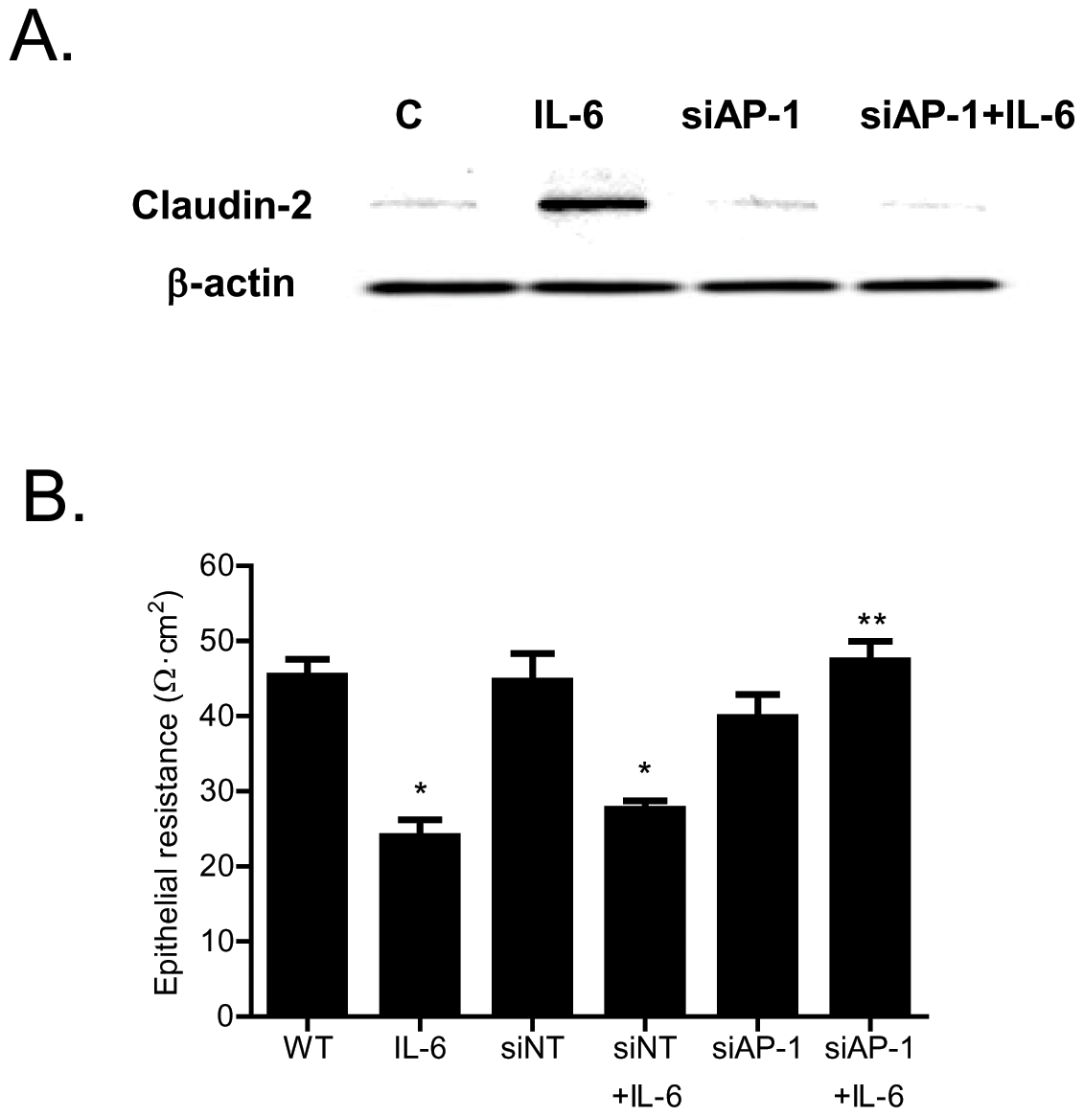
Effect of siRNA induced knock-down of intestinal tissue AP-1 on IL-6 induced drop in intestinal tissue electrical resistance. (A) siRNA induced knock-down of AP-1 *in-vivo* prevented the IL-6 induced increase in mouse intestinal claudin-2 protein expression as assessed by Western blot analysis. B) AP-1 siRNA transfection *in-vivo* prevented the IL-6 induced drop in mouse intestinal TER as measured in Ussing chambers (*n* = 5). _*_, *p*<0.001 vs. control; _**_, *p*<001 vs. IL-6 treatment.

In the following studies, we examined the *in-vivo* relevance of IL-6 effect on mouse intestinal permeability. The effect of intraperitoneal IL-6 administration on mouse intestinal permeability was determined by recycling perfusion of isolated segment (6 cm) of small intestine *in-vivo*, using FITC-labeled dextran (10 Kd) as a paracellular marker [31]. The intestinal tissue resistance was also measured *ex-vivo* by mounting the intestinal tissue in Ussing chamber following IL-6 treatment as described in the Methods section. The intraperitoneal administration of IL-6 (5 µg) at doses used in previous studies [24] did not have significant effect on intestinal permeability to FITC-dextran (Fig. 11A), but caused a 40–50% drop in intestinal tissue electrical resistance (Fig. 11B). The IL-6 effect on intestinal tissue expression of transmembrane TJ proteins claudin-1, claudin-2, claudin-3, claudin-8 and occludin was also examined. IL-6 caused an increase in intestinal tissue claudin-2 expression (Fig. 11C) but did not affect the expression of other transmembrane TJ proteins (data not shown). IL-6 also caused an increase in claudin-2 mRNA expression in mouse intestine (Fig. 11D). Next, the requirement of claudin-2 expression on IL-6 induced drop in intestinal tissue TER was examined. In these studies, intestinal tissue claudin-2 expression was selectively knocked-down by *in-vivo* claudin-2 siRNA transfection of small intestine as described in the Methods section [31]. The siRNA transfection of small intestinal tissue caused a near complete depletion of claudin-2 in the intestinal tissue (Fig. 11E). The intestinal knock-down of claudin-2 prevented the IL-6 induced increase in intestinal claudin-2 expression (Fig. 11E) and drop in intestinal tissue electrical resistance (Fig. 11F), suggesting that the increase in claudin-2 expression was required for the drop in mouse intestinal tissue resistance. Next, we also examined the possible involvement of AP-1 in intestinal claudin-2 expression and intestinal tissue TER. In these studies, the intestinal tissue expression of AP-1 was silenced by *in-vivo* AP-1 siRNA transfection. The siRNA knock-down of AP-1 in mouse small intestine resulted in an inhibition of IL-6 induced increase in claudin-2 expression (Fig. 12A) and drop in intestinal tissue resistance (Fig. 12B).

## Discussion

Interleukin-6 levels are elevated in various inflammatory conditions of the gut including Crohn's disease, ulcerative colitis, and necrotizing enterocolitis [16], [17], [18], and in systemic inflammatory conditions such as sepsis and burn injury [39], [40]. The circulating levels of IL-6 have been reported to range as high as 500 ng/ml in some inflammatory conditions [41]. Although the pro-inflammatory actions of IL-6 on immune system are well-described, the effects on intestinal epithelial barrier remain unclear. Previous studies by Wang et al found that IL-6 causes an enhancement of Caco-2 TJ barrier function through keratin-8 dependent mechanism [22], [42]. IL-6 caused a decrease in trans-epithelial flux of dextran (4 kd) and an increase in keratin-8 level, and inhibition of keratin-8 expression prevented the decrease in dextran-4 kd flux [22], [42]. Consistent with IL-6 barrier enhancing effect, dextran sodium sulfate treatment caused a greater increase in intestinal permeability in IL-6^−/−^ mice than in wild-type mice [22], [42]. IL-6 treatment was also protective against hemorrhagic shock induced intestinal barrier disruption and intestinal inflammation in mice [23]. Additionally, IL-6^−/−^ mice were protected against hemorrhagic shock induced increase in intestinal permeability and bacterial translocation [43]. These studies suggested that IL-6 has an intestinal barrier protective effect. In contrast, Tazuke et al found that IL-6 causes a drop in Caco-2 TER and an increase in mannitol flux in Caco-2 monolayers [25]. Suzuki et al also reported that IL-6 causes a drop in Caco-2 TER but not an increase in dextran (4 kd) flux [24]. Thus, it remains unclear whether IL-6 has a barrier protective or disrupting effect.

The major purpose of this study was to examine and clarify the role of IL-6 on intestinal epithelial TJ barrier. The functional studies in filter-grown Caco-2 monolayers indicated that IL-6 causes a size-selective increase in TJ permeability, such that ionic conductance (assessed by TER) and trans-epithelial flux of small-sized molecule urea (2.9 Å) were increased but not flux of larger sized molecules. The trans-epithelial flux rates of mannitol (4.1 Å), inulin (15 Å), and dextran −10 kd (23 Å) were not affected by IL-6 treatment indicating that IL-6 effect on TJ permeability was size-dependent. IL-6 also did not affect mouse intestinal permeability to large sized molecules but caused a drop in intestinal tissue electrical resistance (Fig. 11B, 11C). The IL-6 effect on Caco-2 TJ permeability was associated with a protein-specific increase in claudin-2 expression (Fig. 2). These findings were consistent with the report by Suzuki et al also showing an increase in claudin-2 expression [24]. Since previous studies have shown that claudin-2 proteins are an essential component of the paracellular pore pathway allowing flux of small-sized molecules having molecular radius <4 Å in size [33], we hypothesized that the IL-6 induced increase in claudin-2 expression was responsible for the size-selective increase in Caco-2 TJ permeability. The claudin-2 knock-down studies confirmed that the increase in claudin-2 was required for the IL-6 increase in Caco-2 TJ permeability. Thus, our data suggested that the IL-6 increase in claudin-2 expression was responsible for the size-selective increase in Caco-2 TJ permeability.

In this study, we also investigated the signaling pathways and the molecular processes involved in IL-6 effect on Caco-2 TJ permeability. As IL-6 is known to activate MAP kinases JNK and p38 kinase, these pathways were considered. Our data showed that IL-6 caused an activation of JNK but not p38 kinase in Caco-2 cells, suggesting that JNK signaling pathway may be involved in IL-6 modulation of TJ permeability. The JNK inhibitor and siRNA silencing studies indicated that JNK activation was required for the increase in claudin-2 expression and TJ permeability. These results suggested that JNK signaling pathway was involved in IL-6 increase in claudin-2 expression and Caco-2 TJ permeability. Additionally, our studies showed that AP-1 was the transcription factor involved in claudin-2 and TJ permeability regulation. Our results indicated that JNK activation leads to AP-1 activation (a direct substrate of JNK) and that AP-1 activation was required for the increase in claudin-2 expression and Caco-2 TJ permeability (Fig. 9). These data suggested that IL-6 induced increase in claudin-2 expression was regulated by JNK activation of AP-1.

The molecular processes involved in IL-6 regulation of claudin-2 expression were also delineated by assessing claudin-2 promoter activity. The increase in claudin-2 protein level directly correlated with the increase in claudin-2 mRNA, suggesting that the increase in claudin-2 level may be causally related to the increase in claudin-2 mRNA transcription. In support of the causal relationship, the inhibition of claudin-2 mRNA transcription with RNA polymerase inhibitor actinomycin-d completely inhibited the IL-6 increase in claudin-2 protein and TJ permeability (Fig. 7). These findings suggested that the increase in claudin-2 expression was due to the increase in claudin-2 gene transcription.

The claudin-2 promoter studies were also carried out to further delineate the molecular processes involved in IL-6 effect on claudin-2 gene activity and TJ permeability. As the claudin-2 minimal promoter region had not been previously reported, the minimal promoter region was determined by progressive deletion of the 5′ end. The promoter deletion studies identified 1170 bp (pCLDN2-C) region as the minimal promoter region (Fig. 8). The IL-6 treatment of Caco-2 monolayers caused an increase in claudin-2 promoter activity. Since AP-1 was found to be a key regulator of claudin-2 protein expression, the involvement of AP-1 in claudin-2 gene regulation was examined. The AP-1 knock-down studies confirmed that AP-1 activation was required for the IL-6 increase in claudin-2 promoter activity and mRNA transcription. In addition, promoter studies using various deletions constructs and site-directed mutagenesis of individual AP-1 binding sites identified the down-stream AP-1 binding motif (H) as the molecular determinant responsible for IL-6 regulation of the claudin-2 promoter activity. Thus, our data suggested for the first time that IL-6 modulation of claudin-2 gene activity and Caco-2 TJ permeability was regulated by binding of the activated AP-1 to the down-stream binding sequence on claudin-2 promoter.

The cell culture studies identified the intracellular signaling pathway and the molecular mechanisms involved in IL-6 regulation of Caco-2 TJ permeability. In this study, we also assessed the *in-vivo* effects of IL-6 on mouse intestinal permeability by re-cycling intestinal perfusion of isolated segment of mouse small intestine. The *in-vivo* perfusion studies indicated that intraperitoneally administered IL-6 does not affect mucosal-to-serosal flux of large molecules but causes a drop in the intestinal tissue electrical resistance. The IL-6 effect on intestinal tissue resistance was similar to IL-6 effect on Caco-2 TER and correlated with the increase in intestinal tissue claudin-2 expression. The intestinal specific knock-down of claudin-2 abolished the IL-6 induced drop in intestinal electrical resistance, confirming that the increase in intestinal tissue claudin-2 expression was responsible for the drop in intestinal tissue resistance. Additionally, AP-1 knock-down studies also showed that IL-6 induced increase in mouse intestinal claudin-2 gene transcript and drop in intestinal tissue electrical resistance were dependent on AP-1 expression. These findings suggested that the IL-6 regulation of mouse intestinal permeability *in-vivo* was also mediated by AP-1 regulation of claudin-2 gene and protein expression.

In conclusion, these studies provide novel insight into the intracellular mechanisms that mediate IL-6 regulation of intestinal epithelial TJ permeability *in-vitro* and *in-vivo*. IL-6 caused a size-selective increase in Caco-2 TJ permeability, which was dependent on claudin-2 gene activation and protein expression. The IL-6 modulation of Caco-2 TJ permeability was regulated by JNK signaling pathway activation of transcription factor AP-1; which, in turn, caused a sequential activation of claudin-2 gene and subsequent increase in gene transcription and protein expression and increase in Caco-2 TJ permeability. Our studies also showed that the IL-6 modulation of mouse intestinal barrier *in-vivo* was regulated by AP-1 dependent increase in claudin-2 expression. Thus, our data show for the first time that IL-6 modulation of intestinal TJ permeability *in-vitro* and *in-vivo* was regulated by JNK activation of AP-1 and AP-1 activation of claudin-2 gene.

## Supporting Information

Figure S1 **Full nucleotide sequence of the cloned claudin-2 promoter region.** Blue highlight indicates the AP-1 binding sequences.
(PDF)
