# Supplementary material for: Interleukin-6 Modulation of Intestinal Epithelial Tight Junction Permeability Is Mediated by JNK Pathway Activation of Claudin-2 Gene
Source: PLoS One. 2014 Mar 24;9(3):e85345. doi: 10.1371/journal.pone.0085345 (PMC3963839; doi:10.1371/journal.pone.0085345)
Supplement: Figure S1 — Full nucleotide sequence of the cloned claudin-2 promoter region. Blue highlight indicates the AP-1 binding sequences. (PDF) [file pone.0085345.s001.pdf]

```
-3179 cacaggctaa tagggaagaa tgagggttta aacaggaaat tgcaacataa tgcaaaatgt
-3119 actatctctg cctcagtttc ttcatctata caatagggat tattagtgcc taccacaaaa
-3059 aaacctgtga cgactaaata agttagcatg taaaaagtgc ttataatggt cccataagaa
                                                   AP-1
-2999 acactacata agtgttgcta ttattattat tactatgatg gaagtaaaca caagatactg
-2939 taagaataga caggaggggt acttgaccca ggctagagta agagacatct gtgctgaatc
-2879 ctaaaagatg agaaggcact aaacagagga agaagaatac ataacactct gcatagaata
-2819 ttctgttcta tttctatttg ggagcaaaag accacatggc atgattgaag aactggaagt
-2759 aatttaattc cacaatagta cagactatga ggagggaaat ggcaagagat gaagcttgag
                                                AP-1
-2699 aggtcatggg gtaaaccaag caagtttgga ctttactttt tagcaaatgg ggagccactg
-2639 gaatgcttta tgtagagtag tgactcgatc agattaatgt gttgaaagat caccttggca
-2579 atgatgtgga gaatgaattg ggagaaagac aagttagaaa gctgttgcta taatccaggt
-2519 gaaagagaat gatgaggtcg tgcatggtgg ctcacacctg taatcccagc attttgggag
-2459 gctgaggtgg gcggatcact tgcagtcagg agttggagat cagcctggcc agcatggcaa
-2399 aaccctgtct ctactaaaaa tacaaaatta gctgggcgtg gtggcgtgca cctgtagtcc
                       AP-1
-2339 cagctactca ggaagctgag gcacaagaat cacttgaacc tgggaggtgg agattgcagt
-2279 gagctaagat cacgccaccc cactccaggc tgggcaacaa aagcaaaatt cggtctcaaa
-2219 aaaagaatgg tgaaactgaa ctagggaagt agcagtggaa atggagagaa aggaaccatg
-2159 gatttgaggt atattcataa ggtagaactg gtgggacata gggactatag gatgcaggag
-2099 gtaagtagaa gaaaggtgct aaagatcact ttccggattt tggtctggca caagtggatg
-2039 gatagaggta tcattcagtg aacacaggga acacagggaa gaactgtttg gggaaagctg
-1979 catgggggat gtgatgagtt cacttttgga catgtcaaat tcgagatgtc tatgagattt
-1919 ccctgtggag attctgacta ggtgattgct ctgccagtct tgaagctcag aagggtctat
-1859 cctggaaatt cagatttggg agggtcagtg gatgggtagt agattaagtt atgggggcaa
-1799 gatgagtttg ctcagggaca gagtgttaat tgagaaaagg atggacttct gaggaatact
-1739 cacatttaag agatgagtag ggaaagagaa atcttgaaga aggctaaaat ggaatagcca
-1679 gattgtggtg ggttgaaaaa ttagtgggtt agttcgggca cggtggctca tgcctgtaat
-1619 cctagcactt tgggaggctg aggcaggtgg atcacttgag cttaggagtt caagaccagt
-1559 gtgggcaaca tggcaaaacc tggtctctac aaaaaataca cacacacaca cacaactagc
-1499 caggtgtggt gatgcgctcc tgtagtccca gctacttgtg gggctgaggc aggaggatca
                                         AP-1
-1439 cacacacaca cacacacaca cacacacaca cacacacaac tagccaggtg tggtgatgcg
-1379 ctcctgtagt cccagctact tgtggggctg aggcaggagg atcacacaca cacacacaca
-1319 cacacacaca cacacacaca caaactagcc aggtgtggtg atgcgctcct gtagtcccag
-1259 ctacttgtgg ggctgaggca ggaggatcac ttgagcccag gaggtcgagg ctgcaatgag
-1199 ccgagatcat gccactgcac tccagcctgg gtgccaaagt gagaacctgt ctcaaaaaga
                       AP-1
-1139 aagaaagaaa aattagtgga tcagagggtc ttgtagagaa tggggaggtg tgcccttgac
                                                  AP-1
-1079 ccttagtgtc ctgaatcttg gcaacaccga gggctccttg aacacggcaa aatcttatat
-1019 ggctctgaga ttccaaagca ttgactcaga tacctgcctc atgcaaagcc ctatattcta
 -959 gagcagtttc cctttcctct gtggcagact cttgtccccc ctaacagatg gcccagggaa
 -899 tttcagggcc ccctctcagt cctggaaccc ttgttccaga gtgctccctc atcatccaag
 -839 aggctgatga tgggagcatc tattaggaga ctggacagga aatgtctggg catgttatac
 -779 atgcaggagg ccttagacta ggctgcagag ggggatttgg gcatggctgg gaggatctga
 -719 actctcagag tatggacaga aggctttgct gcccaccccc atctaccctg gagtagattt
 -659 tcaccatggg cagaatgatc cagggctagg ccactactct ctaggcccct ggagattcaa
 -599 gaggcctcta acaaactgga gtccaagact acattctagg atctgttcct cctgatgtag
 -539 tctgcagttt ggcctcagtc tgcaattgag gggccctatg gcactgttgc ttggcaatgt
 -479 attaaacagc aggccttgga gactagcact tgagttaaca cagccaccac aaccaccact
                                                              AP-1
 -419 gccatcatca ccttcccgga aagcagccac ctgtctggct cctggctttg tccagctgcc
                                                               AP-1
```

```
-359 aacctaaggc atgtgcctac gcaggaggcg atgacatttt ggctccacgt tcaaagttgt
-299 tttttttttc ctttctcatg tgttatttct aaagataaca aaggtcaaaa ggcatccagc
-239 gttttctggt ttctcataag cttctggtca atatttaatc tggtttatgg attttttttta
-179 gGTCTTCTAG ATGCCTTCTT GAGGCTGCTT GTGGCCACCC ACAGACACTT GTAAGGAGGA
-119 GAGAAGTCAG CCTGGCAGAG AGACTCTGAA ATGAGGGATT AGAGGTGTTC AAGGAGCAAG
 -59 AGCTTCAGCC TGAAGACAAG GGAGCAGTCC CTGAAGACGC TTCTACTGAG AGGTCTGCC
```
